# Mouse Maternal High-Fat Intake Dynamically Programmed mRNA m^6^A Modifications in Adipose and Skeletal Muscle Tissues in Offspring

**DOI:** 10.3390/ijms17081336

**Published:** 2016-08-19

**Authors:** Xiao Li, Jing Yang, Youbo Zhu, Yuan Liu, Xin’e Shi, Gongshe Yang

**Affiliations:** 1 Creative Group of Muscle Biology & Genetic Improvement of Pigs, College of Animal Science and Technology, Northwest A&F University, Yangling 712100, China; nice.lixiao@gmail.com (X.L.); yangjing@nwsuaf.edu.cn (J.Y.); zhuyoubo_2015@nwsuaf.edu.cn (Y.Z.); xineshi@nwsuaf.edu.cn (X.S.); 2 College of Life Sciences, Northwest A&F University, Yangling 712100, China; lolily89@outlook.com

**Keywords:** maternal high-fat diet, m^6^A methylation, FTO, METTL3

## Abstract

Epigenetic mechanisms have an important role in the pre- and peri-conceptional programming by maternal nutrition. Yet, whether or not RNA m^6^A methylation—an old epigenetic marker receiving increased attention recently—is involved remains an unknown question. In this study, mouse high-fat feeding prior to conception was shown to induce overweight and glucose intolerant dams, which then continued to be exposed to a high-fat diet during gestation and lactation. The dams on a standard diet throughout the whole experiment were used as a control. Results showed that maternal high-fat intake impaired postnatal growth in male offspring, indicated by decreased body weight and Lee’s index at 3, 8 and 15 weeks old, but the percentages of visceral fat and *tibialis anterior* relative to the whole body weights were significantly increased at eight weeks of age. The maternal high-fat exposure significantly increased mRNA *N*^6^-methyladenosine (m^6^A) levels in visceral fat at three weeks old, combined with downregulated Fat mass and obesity-associated gene (FTO) and upregulated Methyltransferase like 3 (METTL3) transcription, and these changes were reversed at eight weeks of age. In the *tibialis anterior* muscle, the maternal high-fat diet significantly enhanced m^6^A modifications at three weeks, and lowered m^6^A levels at 15 weeks of age. Accordingly, FTO transcription was significantly inhibited at three weeks and stimulated at 15 weeks of age, and METTL3 transcripts were significantly improved at three weeks. Interestingly, both FTO and METTL3 transcription was significantly elevated at eight weeks of age, and yet the m^6^A modifications remained unchanged. Our study showed that maternal high-fat intake could affect mRNA m^6^A modifications and its related genes in offspring in a tissue-specific and development-dependent way, and provided an interesting indication of the working of the m^6^A system during the transmission from maternal nutrition to subsequent generations.

## 1. Introduction

It is well documented that maternal high-fat diet programs tissue development and energy metabolism in offspring [1]. In addition, epigenetic changes such as DNA methylation, resulting from maternal malnutrition, have been demonstrated to play important roles in transgenerational links with metabolic disease [2,3]. *N*^6^-methyladenosine (m^6^A) is currently the most prevalent internal modification of mRNA in eukaryotes, and has been intensively studied recently. An increasing number of studies have shown the fundamental role of m^6^A modification in RNA stability, alternative splicing and translation efficiency, and dysregulation of m^6^A modification may underline a wide range of pathological progressions related to obesity, neurological disorders and male infertility [4].

M^6^A mRNA modification is a highly dynamic process. M^6^A on RNA is believed to be installed by the METTL3 (Methyltransferase like 3)-METTL14-WTAP (Wilms’ tumor 1-associated protein) complex, which is denoted as an m^6^A “writer” [5]. M^6^A modifications can be directly removed by demethylase FTO (Fat mass and obesity-associated protein) [6] and AlkBH5 (AlkB homologue 5) [7], both of which belong to the iron- and 2-oxoglutarate (2OG)-dependent family of AlkB oxygenases [8,9], and are called m^6^A “erasers”. Distinct from AlkBH5, highly expressed in testis and essential for spermatogenesis [7], FTO is most abundant in the hypothalamus and in fat and skeletal muscle, and is strongly related to obesity [10].

The roles of m^6^A modification in the transmission from maternal nutrition to subsequent generations is of great interest, for the m^6^A modification-related genes, especially FTO, are reported to be tightly related with the developmental and metabolic status of adipose tissue and skeletal muscle [10], the two tissues most highly sensitive to maternal nutrition [11,12]. This study is designed to test the effects of maternal high-fat intake on m^6^A modification in adipose and skeletal muscle tissues in the offspring in mice.

## 2. Results

### 2.1. High-Fat Diet Caused Obesity and Glucose Intolerance in Dams

**Figure 1:**
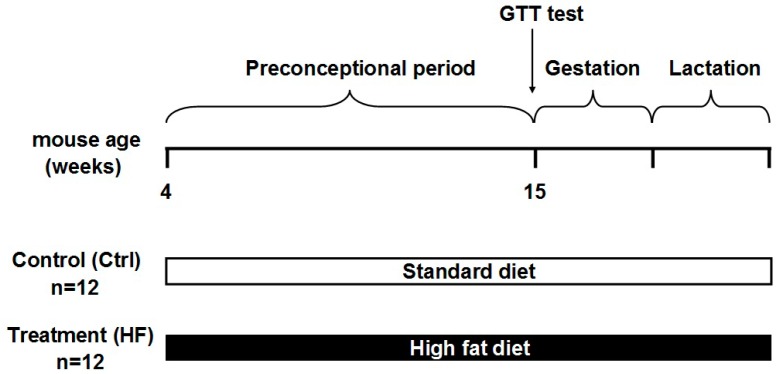
Experimental design. Kunming females were fed a standard or a high-fat diet throughout the whole experiment. Glucose tolerance tests were performed before mating.

**Figure 2:**
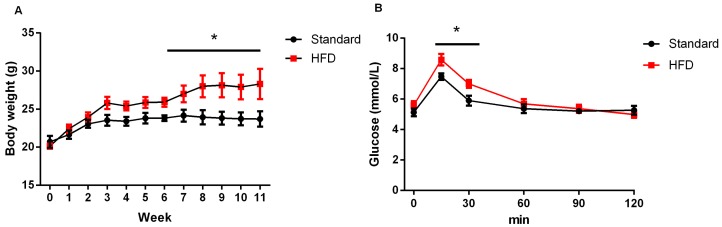
High-fat diet induced overweight (**A**) and glucose intolerance (**B**) in the dams. *n* = 10, * indicates *p* < 0.05.

As shown in Figure 1, dams were fed with either a high-fat diet (45% fat included) or a standard chow diet (10% fat) throughout the whole experiment. Six weeks later, the body weights of female mice fed on a high-fat diet (HFD) became significantly higher than that of their chow-fed counterparts (Figure 2A). In addition, dams in the HFD group showed a slower glucose clearance rate (Figure 2B). At the end of lactation, the body weight of dams in the HFD group were also greatly higher than that of those in the control group (27.95 ± 1.56 vs. 33.18 ± 2.01, *p* < 0.05).

### 2.2. Maternal High-Fat Intake Impaired the Growth of Male Litters

The litter sizes (9.6 ± 0.3 vs. 9.1 ± 0.2, *p* = 0.210) and litter weights (16.80 ± 0.43 vs. 15.85 ± 0.35, *p* = 0.104) were comparable between Standard and HFD dams, and the average birth weights (litter weight/litter size) were also similar (1.75 ± 0.01 vs. 1.74 ± 0.01, *p* = 0.517).

**Figure 3:**
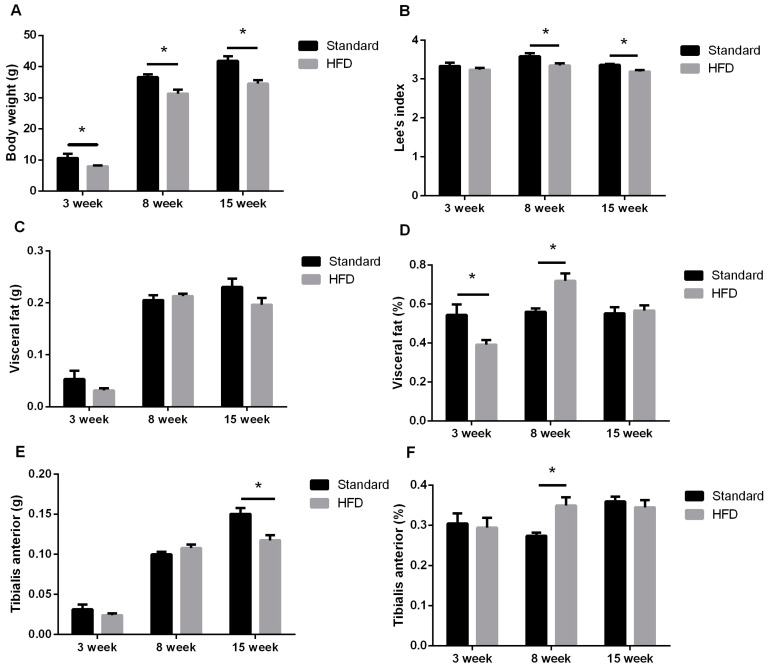
Exposure to maternal high-fat diet resulted in retarded growth in male offspring. Body weights (**A**), Lee’ index (**B**), visceral fat weights (**C**), visceral fat relative weights (**D**), *tibialis anterior* weights (**E**) and *tibialis anterior* relative weights (**F**) have been shown. Lee’s index = [(Body weight (g) × 1000)^1/3^]/Body length (cm); it is an indicator of mouse growth and adiposity. *n* = 10, * indicates *p* < 0.05.

The postnatal weights and Lee’s index of male progeny were significantly reduced after exposure to a maternal high-fat diet (Figure 3A,B). Although the weights of visceral fat tissue were not significantly affected by the maternal diet, the percentage of tissue relative to body weight was significantly decreased at three weeks and increased at five weeks in HFD mice (Figure 3C,D). The weight of *tibialis anterior* muscle significantly declined at 15 weeks (Figure 3E). The reduced weight of *tibialis anterior* muscle at 15 weeks of age might be a result of the retarded body growth, for the percentage of *tibialis anterior* muscle relative to body weight was unchanged (Figure 3F). However, the percentage of *tibialis anterior* muscle relative to body weight increased greatly at eight weeks in the HFD group (Figure 3F).

### 2.3. Maternal High-Fat Diet Altered the m^6^A Pattern in Fat and Skeletal Muscle in a Development-Dependent Way

**Figure 4:**
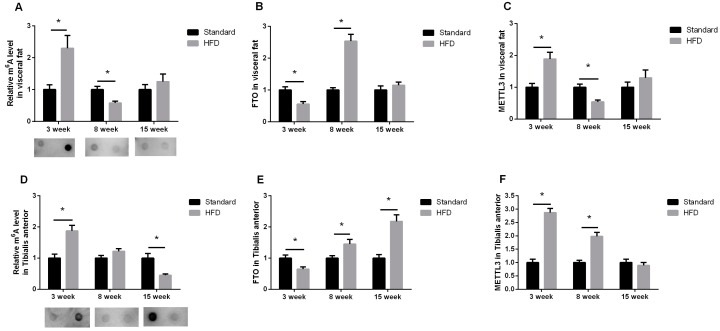
Maternal high-fat intake altered m^6^A methylations and its related genes’ expression in visceral fat and *tibialis anterior* muscle. (**A**–**F**) represent the fold changes of the HFD group relative to the control group of the same age (the means of the control groups were set as one at each timepoint). *n* = 10, * indicates *p* < 0.05.

The effects of a maternal high-fat diet on mRNA m^6^A varied depending on the developmental stage. In visceral fat, mRNA m^6^A levels were significantly increased in three-week-age males, and reduced at eight weeks in the HFD group (Figure 4A). Accordingly, FTO, the “m^6^A eraser”, significantly decreased at three weeks and increased at eight weeks (Figure 4B). The changes of METTL3, the “m^6^A writer”, were opposite to that of FTO (Figure 4C).

As in visceral fat, mRNA m^6^A modifications were significantly higher in the *tibialis anterior* muscle of three-week-age litters in the HFD group (Figure 4D), along with decreased FTO (Figure 4E) and increased METTL3 transcription (Figure 4F). A maternal high-fat diet exerted no significant effects on m^6^A levels in eight-week-age males (Figure 4D), while the expressions of FTO (Figure 4E) and METTL3 (Figure 4F) were simultaneously upregulated. Moreover, exposure to a maternal high-fat diet sharply decreased m^6^A modifications at 15 weeks of age (Figure 4D), accompanied by enhanced FTO expression (Figure 4E), and yet left unchanged the METTL3 expression (Figure 4F).

In 15 week old males, insulin sensitivity was assessed using the homoeostasis model assessment of insulin resistance (HOMA-IR), and no significant differences were observed (*p* > 0.05, Table 1).

## 3. Discussion

It is accepted that maternal nutrition has long-lasting effects on offspring [12,13,14]. In our study, high-fat intake resulted in dams becoming overweight and glucose intolerant (Figure 2), and also impaired the postnatal growth even in adulthood (Figure 3A,B); a result similar to that in a new report on C57BL/6 mice [13]. Interestingly, the weights of visceral fat and *tibialis anterior* muscle relative to total body weight in offspring from HFD dams were significantly higher than that from the control group (Figure 3D,F) at eight weeks old. This increase in adipose and skeletal muscle growth at eight weeks might be a transient compensation for the impaired fetal growth resulting from pre-gestational and gestational exposure to maternal HFD [13].

In our study, the HOMA-IR values between HFD and control groups were comparable, which was inconsistent with previous reports. The difference might be due to different sampling times. In our study, young adults (at 15 weeks old) were used, while much older adults (24 weeks old, or even 12 months old) were studied in other reports [15,16]. Therefore, more time points after 15 weeks would be helpful for evaluating metabolic status in offspring.

Epigenetic mechanisms, such as DNA m^5^C methylation, have been documented to mediate the programming effects of maternal nutrition [3,17]. M^6^A is a predominant mRNA modifier and has gained increasing attention recently [18,19]. M^6^A mRNA modification operates through two main approaches: coordinating protein-RNA interactions, or interacting with m^6^A binding proteins to directly induce RNA splicing, degradation, and translation [4]. M^6^A modifications are developed or “written” by the METTL3-METTL4-WTAP complex [5] and “erased” by FTO [6] or AlkBH5 [7].

In visceral fat, m^6^A methylation in progeny from HFD dams was significantly increased at three weeks and decreased at eight weeks (Figure 4A), which was in striking contrast to the changes of relative visceral fat weight (Figure 3D). There was a similar result indicating that mRNA m^6^A levels downregulate adipogenesis in porcine adipocytes [20]. Other reports show that demethylation of mRNA m^6^A is required for the adipogenesis of the 3T3-L1 preadipocyte cell line [21,22]. Moreover, during the adipogenic process, the demethylation of mRNA m^6^A is exerted by FTO [21,22]. The dynamic regulation of m^6^A by FTO in adipocytes is important in the determination of splicing and transcription of genes contributing to the regulation of adipogenesis (such as RUNX1 translocation partner 1 (RUNX1T1)) [21]. That may be one of the reasons why FTO overexpression promotes obesity [23], and inactive FTO competes with and suppresses obesity [24]. In contrast to FTO, overexpression of METTL3, a critical component of the multiprotein methyltransferase complex for m^6^A methylation [4], could inhibit the expression of pro-adipogenic genes such as PPARγ, and thus suppress adipogenesis and reduce cellular triglyceride content [20]. Taken together, our data show that the m^6^A system may be involved in the adipose tissue development programmed by maternal nutrition.

In *tibialis anterior* muscle, maternal high-fat intake significantly enhanced m^6^A levels at three weeks old and repressed m^6^A modifications at 15 weeks of age. Given the limited reports within our knowledge about mRNA m^6^A in skeletal muscle growth or metabolism, we could not arrive at a clear deduction about the biological significance of the fluctuation of m^6^A modifications in muscle. As for m^6^A related genes, both FTO and METTL3 were simultaneously elevated in the HFD group at eight weeks of age (Figure 4), which was puzzling as their physiological functions are assumed to be contradictory [5,6]. We suppose that this may be due to a compensatory mechanism, for there are indeed several documents describing the mismatch of METTL3 [20] and FTO [25,26] expression with m^6^A levels, especially under physiological conditions where many complicated compensatory pathways exist, and small differences in conditions could affect m^6^A levels.

It is worth mentioning that the expression pattern of FTO is closely matched to the compensatory growth of fat and muscle growth at eight weeks of age in our study, which reminds us of other studies where anabolic pathways were significantly enhanced, catabolism was reduced in abdominal white fat and skeletal muscle of genetically FTO overexpressed (FTO-4) mice [26], and FTO deficiency lead to postnatal growth retardation accompanied by a significant reduction in adipose tissue and lean body mass [24]. In addition to its well-documented pro-adipogenic roles [21,22], FTO also seems to promote muscle growth, for FTO mRNA in muscle was found to increase in the fast growing stages of chickens [27], and FTO transcription is much higher in the breast muscle of fast-growing recessive White Plymouth Rock chickens than that of indigenous Qingyuan partridge chickens at one and eight weeks of age [27].

Of course, the decreased muscle weight with increased FTO expression in 15-week-age progeny argues against the somatotrophic role of FTO discussed above. Mice generally reach body maturation around 10–12 weeks of age, thus we suppose that the subsequent role of FTO in skeletal muscle may be related to energy homeostasis. A previous study has shown that FTO expression is significantly increased in muscles from type 2 diabetic patients, and FTO overexpression could enhance lipogenesis and oxidative stress, reduce the mitochondrial oxidative function, and induce a cluster of metabolic defects associated with type 2 diabetes [28]. The relationship of the elevated FTO expression in muscle with the susceptibility to insulin resistance or type 2 diabetes programmed by maternal high-fat diet [16] should be delicately evaluated in a further study, and more time points after 15 weeks are recommended to better trace the metabolic changes in adult offspring.

Taken together, the current study provides an attractive scenario about the m^6^A landscape during the programming procedure provided by maternal nutrition to the next generation, and the m^6^A system might be a novel and potent mediator from maternal environments to offspring. However, the current study was only focused on the male progeny, and sex-specific responses to maternal over-nutrition have been reported [1,29,30], so that both male and female offspring should be included in the future to better explore the roles of m^6^A system in maternal programming.

## 4. Materials and Methods

### 4.1. Ethics Statement

The study protocol was approved by the Animal Ethics Committee of Northwest A&F University (2014-12-10). Animal handling and sample collection were conducted in accordance with the guidelines of the Management Measures of experimental animals of Shaanxi Province (2011-06-01).

### 4.2. Animals and Diet

Twenty female Kunming mice (purchased from the experimental animal center in The Fourth Military Medical University) around four weeks old (body weight: 20.32 ± 1.78 g) were randomly and equally allocated to one of the two dietary groups: control (Standard) group, receiving the standard laboratory chow diet ad libitum (10% fat, TROPHIC Animal Feed #LAD-0011); and high-fat diet (HFD) group, fed on a high-fat diet ad libitum (45% fat, #TP-0861, TROPHIC Animal Feed High-tech Co., Ltd., Nantong, China). All mice received water freely. Body weight gain was recorded weekly. Eleven weeks later, females were bred with age-matched males and continued on the same diet throughout gestation and lactation. The male mice were fed a standard diet. After weaning, all of the offspring were fed on a standard diet. At 3, 8 and 15 weeks of age, 10 male litters from each group were anesthetized via ether inhalation and killed by bloodletting from the heart. Visceral fat and the *tibialis anterior* muscle were quickly removed and stored in liquid nitrogen.

### 4.3. Glucose Tolerance Test

The glucose tolerance test was carried out in dams at 15 weeks of age (after 11 weeks of experimental diet feeding) after the animals had been denied access to food overnight. Glucose (1 mg/g body weight) was administered to the mice by *intraperitoneal* injection. Tail blood samples were taken before (0 min) and at 30, 60, 90 and 120 min after the glucose administration. The blood glucose levels were determined using an automated blood glucose meter (Sannuo, Changsha, China).

### 4.4. RNA Isolation

Total RNA was extracted by TRIzol Reagent (Invitrogen, Carlsbad, CA, USA), and then treated with DNase I (TaKaRa Bio, Inc., Dalian, China) to remove genomic DNA contamination. Dynabeads^®^ mRNA Purification Kit (Invitrogen, Carlsbad, CA, USA) was used to purify mRNA. Concentrations of RNA were determined with NanoDrop2000 (Thermo Fisher Scientific, Waltham, MA, USA).

### 4.5. RNA Dot-Blot

RNA dot-blot was conducted strictly as previously reported [20]. Firstly, an aliquot of 200 ng mRNA was denatured by heating at 95 °C for 3 min, and immediately cooled down on ice. Aliquot were spotted on nitrocellulose membrane, and subjected to UV cross-linking (1500 × 100 J/cm^2^). The membrane was blocked with 5% of non-fat milk in TBST, and incubated with anti-m^6^A antibody (1:2000, Synaptic Systems, Goettingen, Germany) overnight at 4 °C. After washing 3 times in 1× TBST, the membrane was incubated with anti-rabbit IgG secondary antibody (1:10,000, Boster, Wuhan, China), and visualized by ECL Western Blotting Detection Kit (Thermo, Waltham, MA, USA).

### 4.6. qRT-PCR

Total RNA was subjected to electrophoresis using a 2% agarose gel to verify their integrity. Samples with a 28S/18S rRNA ratio between 1.5 and 2.0 without smears were used for the subsequent RT reaction using a PrimeScript^TM^ RT reagent Kit (TaKaRa Bio, Inc., Dalian, China). qRT-PCR was conducted in technical triplicates with the Multicolor Real-Time PCR detection system (iQ5, Bio-Rad Laboratories, Inc., Hercules, CA, USA). Melting curve analysis was performed at the end of each PCR program to monitor nonspecific product formation. β-actin was used as the internal control. Primers were obtained from PrimerBank (Avaliable online: https://pga.mgh.harvard.edu/primerbank/, PrimerBank ID: 6671509a1), and sequences are given in Table 2.

### 4.7. Insulin Sensitity Assay

Male offspring at 15 weeks old were denied food for 2 h, and blood glucose concentrations (FBG) were determined as described above. Then more blood samples were collected from the heart. The blood samples were left to stand at room temperature for 4 h and then centrifuged at 2000× *g* for 15 min to get serum. Serum insulin concentrations were measured using the Mouse Ultrasensitive ELISA kit (ALPCO, Windham, NH, USA). Insulin sensitivity was assessed using the homoeostasis model assessment of insulin resistance (HOMA-IR). HOMA-IR = FBG (mmol/L) × FIns (mIU/L)/22.5.

### 4.8. Statistical Analysis

All data sets were analyzed with Independent *t* tests by IBM SPSS 20 (Chicago, IL, USA). Results are presented as means ± SEM. Statistical significance was set at *p* < 0.05.

## Figures and Tables

**Table 1:** Homoeostasis model assessment of insulin resistance (HOMA-IR) assay at 15 weeks of age (mean ± SEM).

Groups	FBG (mmol/L)	Fins (mIU/L)	HOMA-IR
Standard group	5.18 ± 0.25	9.76 ± 0.87	2.20 ± 0.15
HFD group	5.67 ± 0.26	10.12 ± 0.68	2.51 ± 0.16

**Table 2:** Primers for real-time polymerase chain reaction (PCR).

Genes	Access No.	Sequences (5′→3′)	Amplicon Size
FTO	NM_011936	F: CCGTCCTGCGATGATGAAGT	119 bp
		R: CCCATGCCGAAATAGGGCTC	
METTLS	NM_019721	F: GAGTTGATTGAGGTAAAGCGAGG	75 bp
		R: GGAGTGGTCAGCGTAAGTTACA	
β-actin	NM_007393	F: GGCTGTATTCCCCTCCATCG	154 bp
		R: CCAGTTGGTAACAATGCCATGT	

## References

[1] Yokomizo H., Inoguchi T., Sonoda N., Sakaki Y., Maeda Y., Inoue T., Hirata E., Takei R., Ikeda N., Fujii M. (2014). Maternal high-fat diet induces insulin resistance and deterioration of pancreatic β-cell function in adult offspring with sex differences in mice. Am. J. Physiol. Endocrinol. Metab..

[2] Wang J., Cao M., Yang M., Lin Y., Che L., Fang Z., Xu S., Feng B., Li J., Wu D. (2016). Intra-uterine undernutrition amplifies age-associated glucose intolerance in pigs via altered DNA methylation at muscle GLUT4 promoter. Br. J. Nutr..

[3] Marco A., Kisliouk T., Tabachnik T., Weller A., Meiri N. (2016). DNA CpG methylation (5mC) and its derivative (5hmC) alter histone post translational modifications at the POMC promoter, affecting the impact of perinatal diet on leanness and obesity of the offspring. Diabetes.

[4] Cao G., Li H.-B., Yin Z., Flavell R.A. (2016). Recent advances in dynamic m^6^A RNA modification. Open Biol..

[5] Liu J., Yue Y., Han D., Wang X., Fu Y., Zhang L., Jia G., Yu M., Lu Z., Deng X. (2014). A METTL3-METTL14 complex mediates mammalian nuclear RNA m^6^-adenosine methylation. Nat. Chem. Biol..

[6] Jia G., Fu Y., Zhao X., Dai Q., Zheng G., Yang Y., Yi C., Lindahl T., Pan T., Yang Y.-G. (2011). *N*6-methyladenosine in nuclear RNA is a major substrate of the obesity-associated FTO. Nat. Chem. Biol..

[7] Zheng G., Dahl J.A., Niu Y., Fedorcsak P., Huang C.-M., Li C.J., Vågbø C.B., Shi Y., Wang W.-L., Song S.-H. (2013). AlkBH5 is a mammalian RNA demethylase that impacts RNA metabolism and mouse fertility. Mol. Cell.

[8] Gerken T., Girard C.A., Tung Y.-C.L., Webby C.J., Saudek V., Hewitson K.S., Yeo G.S.H., McDonough M.A., Cunliffe S., McNeill L.A. (2007). The obesity-associated *FTO* gene encodes a 2-oxoglutarate–dependent nucleic acid demethylase. Science.

[9] Aik W., Scotti J.S., Choi H., Gong L., Demetriades M., Schofield C.J., McDonough M.A. (2014). Structure of human RNA m^6^-methyladenine demethylase AlkBH5 provides insights into its mechanisms of nucleic acid recognition and demethylation. Nucleic Acids Res..

[10] Grunnet L.G., Nilsson E., Ling C., Hansen T., Pedersen O., Groop L., Vaag A., Poulsen P. (2009). Regulation and function of FTO mRNA expression in human skeletal muscle and subcutaneous adipose tissue. Diabetes.

[11] Desai M., Ross M.G. (2011). Fetal programming of adipose tissue: Effects of IUGR and maternal obesity/high fat diet. Semin. Reprod. Med..

[12] MacPherson R.E.K., Castelli L.M., Miotto P.M., Frendo-Cumbo S., Milburn A., Roy B.D., LeBlanc P.J., Ward W.E., Peters S.J. (2015). A maternal high fat diet has long-lasting effects on skeletal muscle lipid and PLIN protein content in rat offspring at young adulthood. Lipids.

[13] Sasson I., Vitins A., Mainigi M., Moley K., Simmons R. (2015). Pre-gestational vs gestational exposure to maternal obesity differentially programs the offspring in mice. Diabetologia.

[14] Gray C., Vickers M.H., Segovia S.A., Zhang X.D., Reynolds C.M. (2015). A maternal high fat diet programmes endothelial function and cardiovascular status in adult male offspring independent of body weight, which is reversed by maternal conjugated linoleic acid (CLA) supplementation. PLoS ONE.

[15] Desai M., Jellyman J.K., Han G., Beall M., Lane R.H., Ross M.G. (2014). Rat maternal obesity and high fat diet program offspring metabolic syndrome. Am. J. Obstet. Gynecol..

[16] Latouche C., Heywood S.E., Henry S.L., Ziemann M., Lazarus R., El-Osta A., Armitage J.A., Kingwell B.A. (2014). Maternal overnutrition programs changes in the expression of skeletal muscle genes that are associated with insulin resistance and defects of oxidative phosphorylation in adult male rat offspring. J. Nutr..

[17] Masuyama H., Mitsui T., Eguchi T., Tamada S., Hiramatsu Y. (2016). The effects of paternal high-fat diet exposure on offspring metabolism with epigenetic changes in the mouse adiponectin and leptin gene promoter. Am. J. Physiol. Endocrinol. Metab..

[18] Dominissini D., Moshitch-Moshkovitz S., Schwartz S., Salmon-Divon M., Ungar L., Osenberg S., Cesarkas K., Jacob-Hirsch J., Amariglio N., Kupiec M. (2012). Topology of the human and mouse m^6^A RNA methylomes revealed by m^6^A-seq. Nature.

[19] Meyer K.D., Saletore Y., Zumbo P., Elemento O., Mason C.E., Jaffrey S.R. (2012). Comprehensive analysis of mrna methylation reveals enrichment in 3′ UTRs and near stop codons. Cell.

[20] Wang X., Zhu L., Chen J., Wang Y. (2015). mRNA m^6^A methylation downregulates adipogenesis in porcine adipocytes. Biochem. Biophys. Res. Commun..

[21] Zhao X., Yang Y., Sun B.-F., Shi Y., Yang X., Xiao W., Hao Y.-J., Ping X.-L., Chen Y.-S., Wang W.-J. (2014). FTO-dependent demethylation of N6-methyladenosine regulates mRNA splicing and is required for adipogenesis. Cell Res..

[22] Zhang M., Zhang Y., Ma J., Guo F., Cao Q., Zhang Y., Zhou B., Chai J., Zhao W., Zhao R. (2015). The demethylase activity of FTO (fat mass and obesity associated protein) is required for preadipocyte differentiation. PLoS ONE.

[23] Church C., Moir L., McMurray F., Girard C., Banks G.T., Teboul L., Wells S., Bruning J.C., Nolan P.M., Ashcroft F.M. (2010). Overexpression of FTO leads to increased food intake and results in obesity. Nat. Genet..

[24] Fischer J., Koch L., Emmerling C., Vierkotten J., Peters T., Bruning J.C., Ruther U., Horsthemke B. (2009). Inactivation of the Fto gene protects from obesity. Nature.

[25] Berulava T., Ziehe M., Klein-Hitpass L., Mladenov E., Thomale J., Rüther U., Horsthemke B. (2013). FTO levels affect RNA modification and the transcriptome. Eur. J. Hum. Genet..

[26] Merkestein M., McTaggart J.S., Lee S., Kramer H.B., McMurray F., Lafond M., Boutens L., Cox R., Ashcroft F.M. (2014). Changes in gene expression associated with Fto overexpression in mice. PLoS ONE.

[27] Song C., Song W.T., Shu J.T., Tao Z.Y., Zhu W.Q., Di C., Li H.F. (2015). Tissue- and breed-specific expression of the chicken fat mass- and obesity-associated gene (FTO). Genet. Mol. Res..

[28] Bravard A., Lefai E., Meugnier E., Pesenti S., Disse E., Vouillarmet J., Peretti N., Rabasa-Lhoret R., Laville M., Vidal H. (2011). FTO is increased in muscle during type 2 diabetes, and its overexpression in myotubes alters insulin signaling, enhances lipogenesis and ros production, and induces mitochondrial dysfunction. Diabetes.

[29] Cunha F.D.S., Dalle Molle R., Portella A.K., Benetti C.D.S., Noschang C., Goldani M.Z., Silveira P.P. (2015). Both food restriction and high-fat diet during gestation induce low birth weight and altered physical activity in adult rat offspring: The “similarities in the inequalities” model. PLoS ONE.

[30] Zhou D., Wang H., Cui H., Chen H., Pan Y.-X. (2014). Early-life exposure to high-fat diet may predispose rats to gender-specific hepatic fat accumulation by programming PEPCK expression. J. Nutr. Biochem..

